# Transurethral plasmakinetic resection versus enucleation for benign prostatic hyperplasia: comparison of intraoperative safety profiles based on endoscopic surgical monitoring system

**DOI:** 10.1186/s12894-022-01014-7

**Published:** 2022-04-19

**Authors:** Qi Jin, En-Guang Yang, Yun-Xin Zhang, Jun Mi, Zhi-Long Dong, Li Yang, Jun-Qiang Tian, Juan Wang, Zhi-Ping Wang

**Affiliations:** 1 Department of Urology, Lanzhou University Second Hospital, Lanzhou, China; 2 Institute of Urology, Lanzhou University Second Hospital, Key Laboratory of Urological Diseases in Gansu Province, Lanzhou, China

**Keywords:** Endoscopic surgical monitoring system (ESMS), Blood loss, Fluid absorption, Transurethral plasmakinetic endoscopic enucleation of the prostate, Transurethral plasmakinetic resection of the prostate

## Abstract

**Objective:**

To compare the intraoperative safety profiles of transurethral plasmakinetic resection of the prostate (PK-TURP) with transurethral plasmakinetic endoscopic enucleation of the prostate (PK-EEP) in the treatment of benign prostatic hyperplasia (BPH) based on endoscopic surgical monitoring system (ESMS).

**Methods:**

A total of 128 patients who were diagnosed with BPH were stratified based on prostate volume (PV) and accepted PK-EEP or PK-TURP treatment at 1:1 ratio. The ESMS as a novel method was used to monitor blood loss and fluid absorption during the operation. Clinical parameters such as intraoperative blood loss volume, fluid absorption volume, operation time, tissue weight of resection, preoperative and postoperative red blood cell count (RBC), hemoglobin concentration (HB), hematocrit (HCT), electrolyte, postoperative bladder irrigation time, indwelling catheter time, hospital stay time and other associated complications were documented and compared between two groups.

**Results:**

No significant differences in majority of baseline characteristics were observed among patients with different prostate volumes between two surgical methods. For patients with prostate volume < 40 ml, the average operation time of patients who received PK-EEP treatment was much more than those who received PK-TURP (*P* = 0.003). On the other hand, for patients with prostate volume > 40 ml, the PK-TURP surgery was associated with a significant increase in intraoperative blood loss (*P* = 0.021, in PV 40–80 ml group; *P* = 0.014, in PV > 80 ml group), fluid absorption (*P* = 0.011, in PV 40–80 ml group; *P* = 0.006, in PV > 80 ml group) and postoperative bladder irrigation time as well as indwelling catheter time but decrease in resected tissue weight compared to the PK-EEP treatment.

**Conclusion:**

The ESMS plays an important role in comparison of intraoperative safety profiles between PK-TURP and PK-EEP. Our data suggest that PK-TURP treatment is associated with a decreased operation time in patients with prostate volume < 40 ml and the PK-EEP treatment is associated with decreased intraoperative blood loss, fluid absorption and increased tissue resection for patients with prostate volume > 40 ml. Our results indicate that the size of prostate should be considered when choosing the right operation method.

## Introduction

Benign prostatic hyperplasia (BPH) is a common disease that mostly occurs in men of advanced ages [1]. A previous study suggested more than half of men with age over 60 years old showed symptoms associated with BPH [2]. As the disease progresses, symptoms such as hematuria, urinary system infection, urinary calculus and bladder decompensation may occur [3]. Currently, many surgical methods are available for the treatment of BPH, such as transurethral resection of the prostate (TURP), simple prostatectomy, transurethral laser vaporization and enucleation [4]. For its superior efficacy and convenience, TURP surgery was ever considered a standard treatment method [5, 6]. However, the intraoperative complications like bleeding and transurethral resection syndrome (TURS) of TURP remain a concern [7].

As a novel treatment method that applied the most advanced plasma bipolar technology [8], the transurethral plasmakinetic resection of the prostate (PK-TURP) can achieve the prostatic incision with thermic energy vaporization as well as consecutive-flow saline irrigation [9]. On the other hand, the transurethral plasmakinetic endoscopic enucleation of the prostate (PK-EEP) is regarded as another novel treatment for BPH [10] which combines the advantages of TURP and open prostatectomy. A clinical study indicated that PK-EEP and PK-TURP had similar efficacy to treat BPH [11]. Unfortunately, the intraoperative safety profiles of these two novel techniques had not been compared, especially in terms of intraoperative blood loss and fluid absorption, because there are currently few accurate, noninvasive and real-time monitoring methods available. To address this problem, we developed the endoscopic surgical monitoring system (ESMS) available worldwide, which can use the physical, mathematical, computer scientific and microelectronic principle to monitor the blood loss and fluid absorption in patients during the endourological operation. The emergence of ESMS solves our inability to predict the risk of TURS with surrogate criteria such as prostate volume, operation time and so on. In addition to this, it allows to assess whether the intraoperative venous sinus opening and surgical capsule perforation are threatening conditions or not through accurate, real-time and noninvasive blood loss and fluid absorption monitoring. The accuracy of this method has been validated in previous study [12]. To provide a better reference for BPH management, we designed this prospective study and compared the intraoperative safety profiles of these two novel treatment methods based on ESMS.

## Methods

### Patients

A total of 128 patients diagnosed with BPH at our department from June 2020 to March 2021 were enrolled in this study and received PK-EEP (n = 65) or PK-TURP (n = 63) treatment. All screened patients received routine preoperative evaluation such as whole blood cell count, electrolyte, urine examination, urine flow rate, liver and kidney function and prostate specific antigen (PSA) level. The prostate volume was determined by preoperative transrectal ultrasound. The inclusion criteria were as follows: (1) patients with age ≥ 50, (2) Qmax ≤ 10 ml/s, (3) IPSS ≥ 8 or at least one history of acute urinary retention, (4)ineffective drug treatment or repeated urinary tract infection. The exclusion criteria were as follows: (1) patients with cardiopulmonary insufficiency, (2) liver insufficiency, (3) renal insufficiency, (4) severe abnormal blood coagulation, (5) collection of irrigation fluid leakage, (6) complicated with bladder stone. All patients were stratified according to the prostate volume: < 40 ml, 40–80 ml, > 80 ml. This study was approved by the Ethics Committee of the Second Hospital of Lanzhou University (2021A-180) and all patients signed the informed consent.

### Operation monitor

The endoscopic surgical monitoring system (Aokai Medical Equipment Co. Ltd, GanSu, China) was used to measure blood loss and fluid absorption during the operation. It had been approved by Chinese Food and Drug Administration for manufacture and clinical application (approved number: 20162210011). The ESMS was composed of irrigating liquid input measurement module (high-precision weight sensor and non-contact flow meter), blood measurement probe (photoelectric probe), liquid collection system (liquid collection barrel), intelligent collection and monitoring module, computer software analysis system and liquid crystal display panel. Our team has previously demonstrated the validity and accuracy of this system [12]. Before surgery, we usually entered the information about patient such as ID number, operation type, preoperative hemoglobin concentration, age, infusion type, height, weight, irrigation fluid type on liquid crystal display of ESMS. When the operation began, we would click the “start” button on liquid crystal display of ESMS. At every moment of the operation, the blood loss volume and fluid absorption volume could be obtained based on ESMS. When the operation came to an end, we could get the final blood loss and fluid absorption volume. During the operation, according to our department experiences, the ESMS was set to alert the operator when the blood loss volume was greater than 300 ml or fluid absorption volume was more than 1500 ml.

### Surgical procedures

All operations were performed by three same skilled and experienced urologists. They all have done PK-EEP and PK-TURP more than 800 surgeries respectively before the participation in this study. Besides they followed the same technique, every urologist roughly conducted 21PK-TURP operations and 21 PK-EEP operations. The independent researcher is set up to avoid liquid spillage on the floor. The plasma bipolar electroresection system (Olympus Corp, Tokyo, Japan) was applied in surgery. The power was set as 280 W for cutting and 180 W for coagulation in these two surgical methods. Saline solution was used as the irrigation solution (3000 ml, QingshanLikang Pharmaceutical, Chengdu, China). All patients received prophylactic antibiotics and were anesthetized with lumbar anesthesia before surgery began. Patients were placed at lithotomy position and received standard operation preparation.

In PK-TURP, the procedure employed the acknowledged steps of transurethral resection. A marker sulcus was cut from the bladder neck to the proximal verumontanum at points 5 and 7. With this marker sulcus as a sign, the hyperplasia tissue was gradually excised by the electric ring. The middle lobe was excised, and then the left lobe was excised. The right lobe was treated in the same way until the prostate adenoma was completely removed.

The PK-EEP procedure was similar as Liu et al. [10]. In PK-EEP, the middle lobe of prostate was peeled by the cutting ring at points 5 and 7 O’clock. Then we continued to peel off the bilateral lobe of prostate in the direction of 7 to 12 O’clock respectively and converged at 12 O’clock. Subsequently, the junction between the gland and the bladder neck urethral mucosa was dissected. From the apex of the prostate to the bladder neck direction, the prostatic surgical capsule was expanded to the depth of the existing layer until hyperplastic adenoma tissue was completely peeled. Furthermore, the peeled prostate adenoma tissues were pushed into the bladder and shattered with morcellator (Hawk corp, Hangzhou, China).

### Statistical analysis

SPSS 26.0 software (IBM Corp, Armonk, NY) was used for all statistical analysis. All data were expressed as mean ± standard deviation or medians with interquartile ranges or cases (%). The two independent Student's t test was used to analyze data conformed to the normal distribution. Mann–Whitney U test was used when the data did not fit the normal distribution. Enumeration data was tested by Pearson's Chi-square test or Fisher's exact test. Then, multivariate linear regression was used to identify the factors for predicting intraoperative fluid absorption volume. *P* < 0.05 was identified as statistically significant.

## Results

**Fig. 1:**
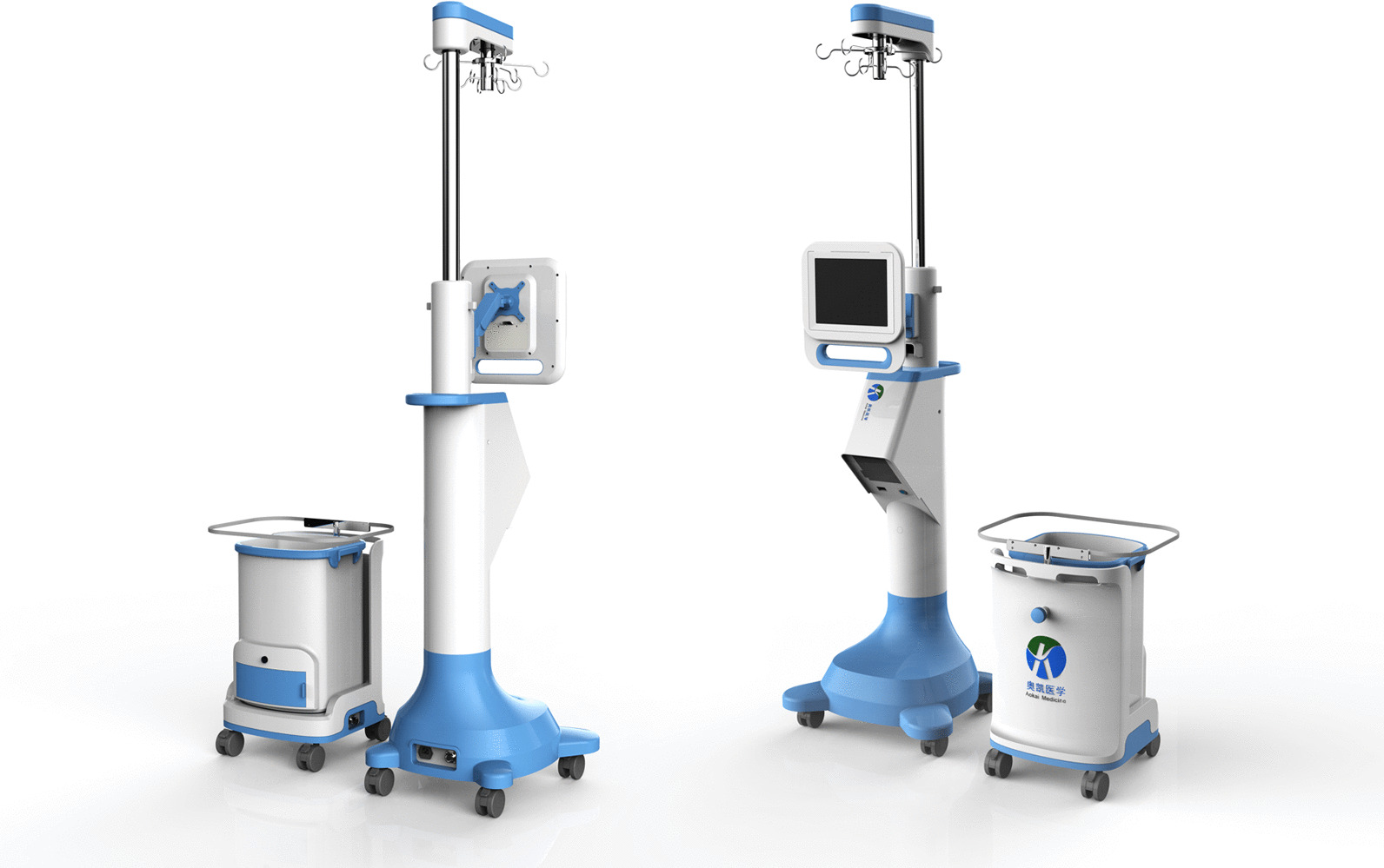
The schematic diagram of endoscopic surgical monitoring system

**Fig. 2:**
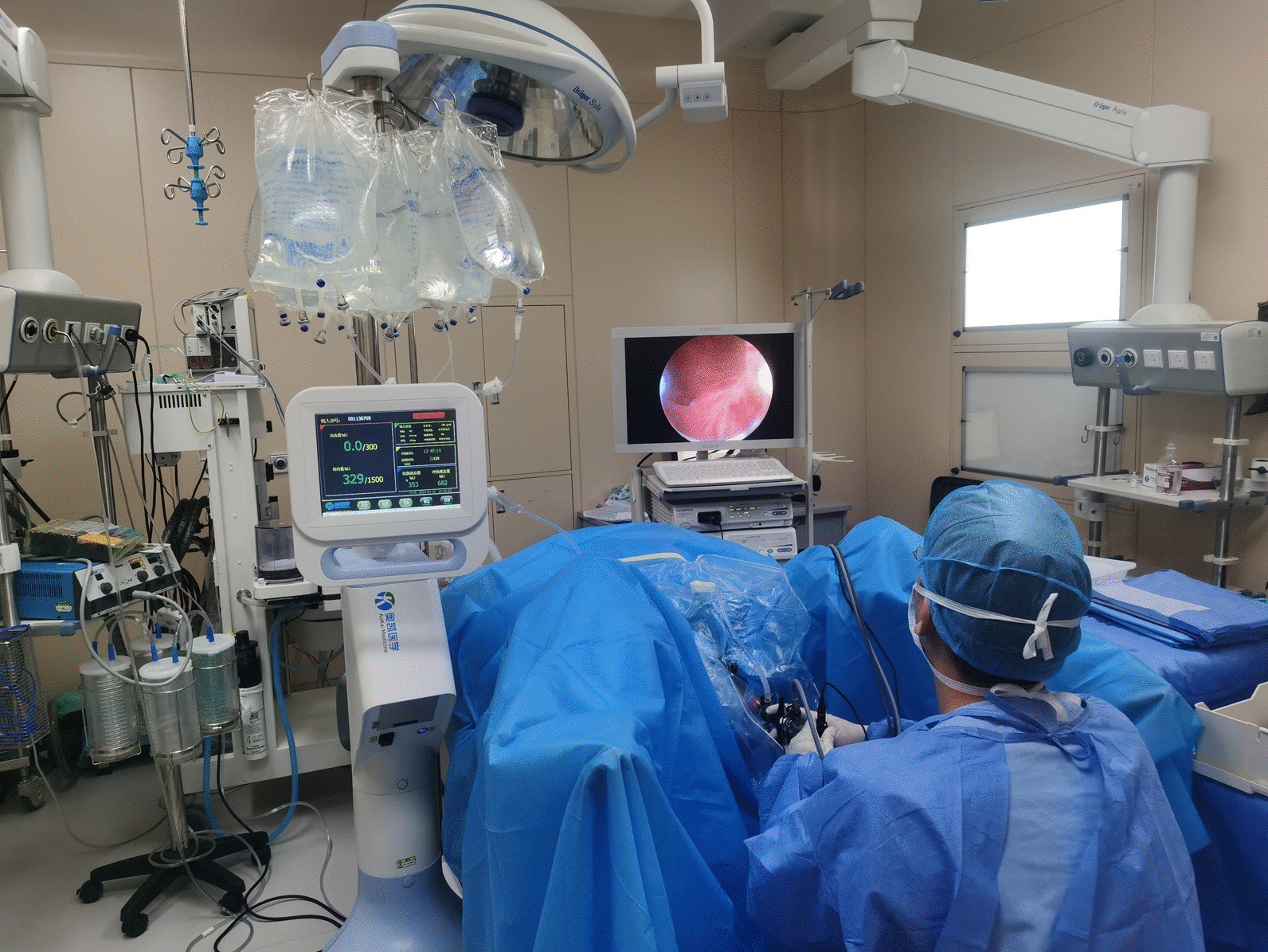
Illustrations depicting intraoperative condition and application of endoscopic surgical monitoring system

No significant differences in majority of baseline parameters were identified in patients who received PK-TURP surgery or PK-EEP surgery at any prostate volume (Table 1). The ESMS and the intraoperative situation were shown in Figs. 1, 2, respectively. All operations were successfully carried out and no patient experienced blood transfusion, TURS and severe postoperative complications. There was no difference in postoperative complications of patients who received PK-TURP or PK-EEP at any prostate volume (Table 2).

**Table 1:** Preoperative baseline data of all patients

Variable	PV < 40 ml			40 ml ≤ PV ≤ 80 ml			PV > 80 ml		
	PK-TURP (n = 15)	PK-EEP (n = 19)	*P-value*	PK-TURP (n = 37)	PK-EEP (n = 33)	*P-value*	PK-TURP (n = 11)	PK-EEP (n = 13)	*P-value*
Age (year)	72.67 ± 7.99	66.37 ± 6.14	**0.014**	71.11 ± 7.4	70.61 ± 5.49	0.751	72.64 ± 6.76	72.23 ± 10.96	0.916
BMI (kg/m^3^)	22.57 ± 2.4	23.32 ± 4.35	0.527	23.5 ± 3.88	22.9 ± 2.3	0.434	23.62 ± 3.22	24.56 ± 3.21	0.483
TPSA (ng/ml)	2.32 (1.22–8.55)	1.39 (0.70–2.49)	0.155	4.86 (2.63–8.02)	5.08 (2.35–8.12)	0.787	7.86 ± 4.49	6.89 ± 3.93	0.576
FPSA (ng/ml)	0.47 (0.25–1.24)	0.33 (0.12–0.51)	0.107	0.89 (0.58–1.76)	0.84 (0.49–1.37)	0.437	1.81 ± 1.14	1.61 ± 0.94	0.640
RBC (X10^12^)	4.73 ± 0.61	4.85 ± 0.54	0.570	4.7 ± 0.54	4.77 ± 0.71	0.673	5.13 ± 0.65	4.62 ± 0.58	0.050
HB (g/L)	144.87 ± 17.74	153.32 ± 15.88	0.153	147.73 ± 17.08	148.52 ± 17.72	0.851	153.91 ± 26.21	146.31 ± 18.56	0.416
HCT (L/L)	0.44 ± 0.05	0.46 ± 0.05	0.212	0.44 ± 0.05	0.45 ± 0.05	0.569	0.47 ± 0.07	0.44 ± 0.05	0.239
K^+^ (mmol/L)	3.87 ± 0.31	3.92 ± 0.32	0.675	3.91 ± 0.4	4.02 ± 0.56	0.350	3.75 ± 0.31	3.71 ± 0.52	0.836
Na^+^ (mmol/L)	139.53 ± 3.36	140.47 ± 3.17	0.406	139.71 ± 3.34	140.82 ± 3.74	0.192	140.09 ± 2.55	142 ± 2.94	0.107
Cl^−^ (mmol/L)	100.91 ± 3.56	102.02 ± 3.63	0.377	101.11 ± 4.05	102.61 ± 4.00	0.125	101.27 ± 1.85	104 ± 3.94	**0.039**
Ca^2+^ (mmol/L)	2.21 ± 0.13	2.25 ± 0.12	0.322	2.24 ± 0.16	2.21 ± 0.11	0.457	2.15 ± 0.08	2.15 ± 0.08	0.920
Mg^2+^ (mmol/L)	0.89 ± 0.07	0.89 ± 0.06	0.825	0.87 ± 0.1	0.88 ± 0.06	0.421	0.9 ± 0.07	0.89 ± 0.08	0.689

Data is presented as means ± standard deviations or medians with interquartile ranges

The significance of bold means *P-value* < 0.05

*PV*, prostate volume; *PK-TURP*, transurethral plasmakinetic resection of the prostate; *PK-EEP*, transurethral plasmakinetic endoscopic enucleation of the prostate; *BMI*, body mass index; *TPSA*, total prostate specific antigen; *FPSA*, free prostate specific antigen; *RBC*, red blood cell count; *HB*, hemoglobin; *HCT*, haematocrit

**Table 2:** Comparison of postoperative complications in all groups

Variable	PV < 40 ml			40 ml ≤ PV ≤ 80 ml			PV > 80 ml		
	PK-TURP (n = 15)	PK-EEP (n = 19)	*P-value*	PK-TURP (n = 37)	PK-EEP (n = 33)	*P-value*	PK-TURP (n = 11)	PK-EEP (n = 13)	*P-value*
*Clavien-Dindo I*									
Bladder spasm	2 (13.33)	1 (5.26)	0.571	6 (16.22)	4 (12.12)	0.883	5 (45.45)	2 (15.38)	0.182
Fever	0	0	—	1 (2.7)	1 (3.03)	1	0	0	—
Hypothermia	0	0	—	0	0	—	1 (9.09)	0	0.458
*Clavien-Dindo II*									
Blood transfusion	0	0	—	0	0	—	0	0	—
Clot retention	3 (20)	2 (10.53)	0.634	7 (18.92)	5 (15.15)	0.676	2 (18.18)	1 (7.69)	0.576
Transient incontinence	1 (6.67)	4 (21.05)	0.355	4 (10.81)	2 (6.06)	0.779	3 (27.27)	2 (15.38)	0.630
*Clavien-Dindo IIIa*									
Secondary hemorrhage	0	0	—	0	0	—	0	0	—
Urethral stricture	0	0	—	1 (2.7)	2 (6.06)	0.919	1 (9.09)	0	0.458
TURS	0	0	—	0	0	—	0	0	—

Data is presented as cases (%)

*TURS*, transurethral resection syndrome

The intraoperative parameters were summarized and presented in Table 3. Our data suggested among patients with prostate volume < 40 ml, the average operation time was significantly increased in those who were assigned to the PK-EEP group compared to the PK-TURP group (75.47 ± 26.12 min vs. 49.27 ± 20.09 min, *P* = 0.003). Meanwhile, no significant differences of intraoperative blood loss volume (*P* = 0.182), fluid absorption volume (*P* = 0.208), tissue resection weight (*P* = 0.508) and venous sinus opening (*P* = 0.187) were observed between two groups. Interestingly, we noticed that in patients with prostate volume 40–80 ml and > 80 ml, the difference of operation time was not discovered but the PK-TURP was associated with increased intraoperative blood loss volume (118.2 (77.3–167.6) ml vs. 90 (54.85–120) ml, *P* = 0.021, in PV 40–80 ml group; 209.68 ± 98.95 ml vs. 126.43 ± 49.66 ml, *P* = 0.014, in PV > 80 ml group), fluid absorption volume (947.08 ± 298.74 ml vs. 775.38 ± 246.92 ml, *P* = 0.011, in PV 40–80 ml group; 1169.45 ± 264.42 ml vs. 872.08 ± 219.62 ml, *P* = 0.006, in PV > 80 ml group), yet decreased in resected tissue weight (37.4 ± 7.75 g vs. 42.4 ± 10.3 g, *P* = 0.025, in PV 40–80 ml group; 68.39 ± 17.78 g vs. 84.18 ± 13.78 g, *P* = 0.027, in PV > 80 ml group). Additionally, venous sinus opening occurred more frequently in patients with PK-TURP than PK-EEP for prostate volume 40–80 ml (9 (24.32%) vs. 0 (0), *P* = 0.007).

**Table 3:** Comparison of intraoperative parameters in all groups

Variable	PV < 40 ml			40 ml ≤ PV ≤ 80 ml			PV > 80 ml		
	PK-TURP (n = 15)	PK-EEP (n = 19)	*P-value*	PK-TURP (n = 37)	PK-EEP (n = 33)	*P-value*	PK-TURP (n = 11)	PK-EEP (n = 13)	*P-value*
Operation time (min)	49.27 ± 20.09	75.47 ± 26.12	**0.003**	73.22 ± 25.72	81.76 ± 25.93	0.172	93.45 ± 25.65	99.38 ± 27.2	0.591
Resected tissue weight (g)	14.25 ± 5.16	15.5 ± 5.57	0.508	37.4 ± 7.75	42.4 ± 10.3	**0.025**	68.39 ± 17.78	84.18 ± 13.78	**0.027**
Blood loss (ml)	43.6 (20–78.3)	82 (32.1–108)	0.182	118.2 (77.3–167.6)	90 (54.85–120)	**0.021**	209.68 ± 98.95	126.43 ± 49.66	**0.014**
Fluid absorption (ml)	578.23 ± 264.38	692.58 ± 252.78	0.208	947.08 ± 298.74	775.38 ± 246.92	**0.011**	1169.45 ± 264.42	872.08 ± 219.62	**0.006**
Venous sinus opening [cases (%)]	2 (13.33)	0 (0)	0.187	9 (24.32)	0 (0)	**0.007**	3 (27.27)	2 (15.38)	0.630
Surgical capsule perforation [cases (%)]	0 (0)	0 (0)	—	1 (2.7)	0 (0)	1	2 (18.18)	0 (0)	0.199

Data is presented as means ± standard deviations or medians with interquartile ranges or cases (%)

The significance of bold means *P-value* < 0.05

Table 4 described the postoperative data of patients who received different treatments. For patients with prostate volume < 40 ml, it appeared that no significant difference was observed between the PK-EEP and PK-TURP groups. However, among patients with prostate volume 40–80 ml and > 80 ml, the average postoperative bladder irrigation time (1.5 (1–2) days vs. 2 (1.25–3) days, *P* = 0.025, in PV 40–80 ml group; 1 (1–1.75) days vs. 2.5 (1.5–2.5) days, *P* = 0.007, in PV > 80 ml group) and indwelling catheter time (3.59 ± 1.16 days vs. 4.46 ± 1.39 days, *P* = 0.006, in PV 40–80 ml group; 3 (2.5–3.75) days vs. 4.5 (3.5–6.5) days, *P* = 0.001, in PV > 80 ml group) of patients in the PK-EEP group significantly decreased when compared to the PK-TURP group. In addition, for patients with prostate volume > 80 ml, the postoperative hospitalization time was significantly increased among patients that received PK-TURP surgery compared to those who received PK-EEP surgery (5.5 (4–7) days vs. 3.5 (3.25–5) days, *P* = 0.008).

**Table 4:** Comparison of postoperative parameters in all groups

Variable	PV < 40 ml			40 ml ≤ PV ≤ 80 ml			PV > 80 ml		
	PK-TURP (n = 15)	PK-EEP (n = 19)	*P-value*	PK-TURP (n = 37)	PK-EEP (n = 33)	*P-value*	PK-TURP (n = 11)	PK-EEP (n = 13)	*P-value*
RBC (X10^12^)	4.67 ± 0.59	4.66 ± 0.48	0.924	4.62 ± 0.66	4.65 ± 0.6	0.852	5.02 ± 0.76	4.51 ± 0.65	0.090
Hb (g/L)	140.8 ± 18.49	145.58 ± 15.32	0.416	143.05 ± 21.23	143.7 ± 17.16	0.890	151.27 ± 29.63	141 ± 20.49	0.328
HCT (L/L)	0.43 ± 0.05	0.44 ± 0.04	0.592	0.43 ± 0.06	0.43 ± 0.05	0.877	0.46 ± 0.08	0.42 ± 0.07	0.233
K^+^ (mmol/L)	3.83 ± 0.42	3.96 ± 0.52	0.419	3.98 ± 0.53	3.79 ± 0.44	0.104	3.65 ± 0.27	3.83 ± 0.48	0.282
Na^+^ (mmol/L)	139.07 ± 3.41	139.57 ± 3.97	0.701	138.51 ± 2.67	139.16 ± 2.69	0.315	140.36 ± 3.46	140.71 ± 3.37	0.803
Cl^−^ (mmol/L)	101.94 ± 3.73	102.84 ± 3.82	0.497	101.41 ± 3.21	102.66 ± 4.06	0.156	103.07 ± 4.12	103.67 ± 3.44	0.703
Ca^2+^ (mmol/L)	2.2 ± 0.15	2.19 ± 0.17	0.839	2.21 ± 0.17	2.23 ± 0.11	0.562	2.19 ± 0.16	2.13 ± 0.2	0.435
Mg^2+^ (mmol/L)	0.81 ± 0.1	0.82 ± 0.06	0.576	0.82 ± 0.1	0.8 ± 0.07	0.534	0.79 ± 0.05	0.79 ± 0.07	0.819
Postoperative bladder irrigation time (d)	1.47 ± 0.58	1.82 ± 0.89	0.197	2 (1.25–3)	1.5 (1–2)	**0.025**	2.5 (1.5–2.5)	1 (1–1.75)	**0.007**
Postoperative indwelling catheter time (d)	4.63 ± 1.46	4.05 ± 1.18	0.208	4.46 ± 1.39	3.59 ± 1.16	**0.006**	4.5 (3.5–6.5)	3 (2.5–3.75)	**0.001**
Postoperative hospital stay time (d)	4.93 ± 1.49	4.45 ± 1.25	0.307	4.57 ± 1.33	4.49 ± 1.21	0.787	5.5 (4–7)	3.5 (3.25–5)	**0.008**

Data is presented as means ± standard deviations or medians with interquartile ranges

The significance of bold means *P-value* < 0.05

Table 5 described a multivariate linear regression model for some factors predicting intraoperative fluid absorption volume. The significant predictors for intraoperative fluid absorption were considered as surgery type (*P* < 0.001, 95% CI − 238.32 to − 92.59), operation time (*P* < 0.001, 95% CI 5.28–8.1), venous sinus opening or surgical capsule perforation (*P* < 0.001, 95% CI 100.31–312.62) and prostate volume (*P* = 0.045, 95% CI 0.03–2.75).

**Table 5:** Multivariate linear regression model for some factors predicting intraoperative fluid absorption

	Predicted Model: F (6,121) = 38.64, *p* < 0.001				
	R = 0.81, R^2^ = 0.66, Adjusted R^2^ = 0.64				
	Unstandardized coefficients		Standardized coefficients		
	B	SE	β	*p*-value	95%CI for β
Constant	668.00	197.28		0.001*	277.44to1058.57
Surgery type 0 = PK-TURP, 1 = PK-EEP	− 165.45	36.81	− 0.27	< 0.001*	− 238.32to − 92.59
Operation time (min)	6.69	0.71	0.62	< 0.001*	5.28to8.10
VSO + SCP 0 = None, 1 = Yes	206.46	53.62	0.24	< 0.001*	100.31to312.62
Prostate volume	1.39	0.69	0.13	0.045*	0.03to2.75
Age	− 3.01	2.25	− 0.07	0.182	− 7.46to1.43
BMI	− 6.72	4.98	− 0.07	0.180	− 16.58to3.14

*Significance at *p*-value < 0.05

*R*, multiple correlation coefficient; *CI*, confidence interval; *min*, minute; *VSO + SCP*, venous sinus opening or surgical capsule perforation

## Discussion

To the best of our knowledge, this is the first study designed to compare the intraoperative safety profiles of the PK-EEP treatment and the PK-TURP treatment for BPH based on endoscopic surgical monitoring system (ESMS). We did not randomize patients because the patients needed to sign informed consent forms and made a decision on which surgical method was conducted referring to the surgeon's advice before surgery. However, we could ensure all operations were performed by same skilled and experienced urologists, the majority of baseline data for patients were comparable, all clinical data were collected prospectively and the independent researcher did not know which operation method the patients received before surgery. Although the bipolar technology reduces the risk of TURS with normal saline as irrigating fluid, fluid overload is also a serious life-threatening complication due to a large amount of irrigation fluid absorption through prostatic vessels and extravascular routes [13–15]. Some factors may influence irrigation fluid absorption, such as prostate volume, surgical method, operation time, venous sinus opening, capsular perforation [14]. Once a large amount of normal saline irrigation fluid absorption has occurred, acute fluid overload and hyperchloremic acidosis will emerge [13, 16]. It is worth noting that the normal saline is not matched to human physiology, the chlorine concentration of normal saline is higher than human [13]. In addition, acute fluid overload and hyperchloremic acidosis may lead to some more serious consequences, for example, impaired myocardial contractility, hypotension, decrease diuresis, pulmonary oedema and so on [13]. These complications can be life-threatening to patients. Therefore, it is very important to monitor the absorption of saline solution during the operation.

TURS is a life-threatening condition manifesting as neural and cardiovascular symptoms due to the disturbance of body fluid and electrolytes homeostasis which is often induced by absorption of abundant irrigation fluid. Therefore, it is important to monitor the absorption volume of the irrigation fluid [17]. Some researchers [17–20] used alcohol as a marker to assess the volume of fluid absorption throughout the TURP operation. However, there is a delay for ethanol to diffuse into the circulatory system which might result in misrepresentation of the situation [20]. In addition, some patients may be allergic to ethanol. For another, intraoperative bleeding has always been a serious problem in surgery process [21]. Nevertheless, many previous studies used a simple formula to estimate intraoperative blood loss volume [6, 11, 22, 23], which might not accurately reflect the real blood loss volume. To better evaluate the blood loss and fluid absorption, the endoscopic surgical monitoring system was developed and used in BPH surgeries. On the one hand, it can utilize blood measurement probe to monitor hemoglobin concentration (HB) in liquid collection barrel, then through comparison with the preoperative HB of patients, the blood loss volume can be drawn. On the other side, the fluid absorption volume can be calculated based on fluid input volume, estimated urine output volume, blood loss volume, and fluid output volume by irrigating fluid measurement module. ESMS has met the aims of measuring blood loss and fluid absorption concurrently, accurately and noninvasively in real time during the operation process [12]. Beyond that, the ESMS can also alert the surgeon in time, when the fluid absorption volume is beyond 1000 ml in display, then the patient is administered Furosemide (20 mg) via intravenous injection immediately. Once the fluid absorption volume exceeds 1500 ml, the operation will be stopped in time to ensure the patient's safety. Moreover, when the blood loss volume is more than 300 ml, the blood transfusion therapy will be considered.

The head-to-head study of PK-TURP surgery and PK-EEP surgery can provide the reference for selection of appropriate surgical treatment for BPH. It was interesting to discover the patients with different prostate volumes responded differently to the PK-EEP surgery and PK-TURP surgery in this study. For patients with relatively small prostate volume (< 40 ml), the blood loss, fluid absorption and the resected tissue weight were comparable between two methods. As the steps of PK-TURP are simpler than PK-EEP, the operation time was decreased in patients who received the PK-TURP surgery. Meanwhile, for patients with relatively large prostate volume (40–80 ml or > 80 ml), the advantage of PK-TURP in reducing operation time disappeared but was associated with significantly increased intraoperative blood loss and fluid absorption as well as some intraoperative complications. These observations were similar to previous study (Luo et al. [11]), which showed that the blood loss during PK-EEP surgery was decreased compared to PK-TURP in patients with large volume prostate. But the strength of our study is monitoring the blood loss accurately by ESMS. It is important to point out that the study by Ran et al. that suggested no difference between PK-EEP and PK-TURP in terms of fluid absorption [17] failed to stratify the patients based on their prostate volume. The benefits of applying PK-EEP in patients with relatively large prostate volume might be due to the fact that the hyperplastic prostate tissue was peeled off directly along the surgical capsule which could remove more tissue and avoided excessive opening venous sinus in prostate [6]. So, in our data, for prostate volume > 40 ml with PK-EEP, more tissues were removed, and the blood loss and fluid absorption volume through the intravascular pathway were decreased, therefore reduced the postoperative bladder irrigation and catheterization indwelling period.

However, this study still had some limitations. Firstly, this was a prospective non-randomized concurrent controlled study conducted in a single center. Even though with a relatively large cohort, it still might not be representable to the general population. Secondly, our study mainly focused on the intraoperative safety profiles, a longer follow-up period was necessary to better understand the benefits and drawbacks of these two surgical methods. Furthermore, this study was not designed to examine the differences of efficacy between these two operations.

## Conclusion

This study indicates there are no differences in intraoperative blood loss and fluid absorption between PK-TURP and PK-EEP for patients with prostate volume < 40 ml, but PK-TURP is associated with a decreased operation time. For patients with prostate volume > 40 ml, PK-EEP should be considered due to its ability to decrease blood loss, fluid absorption, perioperative recovery time and enucleate more tissue. Finally, ESMS is an accurate and effective method in monitoring blood loss and fluid absorption, which is beneficial for improving the intraoperative safety profiles during transurethral prostate resection procedures.

## Data Availability

All data analyzed during this study are included in this article. The data used to support the findings of this study are available from the corresponding author upon request.

## Abbreviations

PK-TURP: Transurethral plasmakinetic resection of the prostate
PK-EEP: Transurethral plasmakinetic endoscopic enucleation of the prostate
BPH: Benign prostatic hyperplasia
ESMS: Endoscopic surgical monitoring system
RBC: Red blood cell count
HB: Hemoglobin concentration
HCT: Hematocrit
TURP: Transurethral resection of the prostate
PSA: Prostate specific antigen
TURS: Transurethral resection syndrome
PV: Prostate volume
VSO: Venous sinus opening
SCP: Surgical capsule perforation
